# New Cable Delay Measurement System for VGOS Stations

**DOI:** 10.3390/s22062308

**Published:** 2022-03-16

**Authors:** Pablo García-Carreño, Javier González-García, María Patino-Esteban, Francisco J. Beltrán-Martínez, Marta Bautista-Durán, Pablo Luis López-Espí, José A. López-Pérez

**Affiliations:** 1 Yebes Observatory, Centro de Desarrollos Tecnológicos, Instituto Geográfico Nacional, Ministerio de Transportes, Movilidad y Agenda Urbana, Cerro de la Palera s.n., 19141 Yebes, Spain; j.gonzalez@oan.es (J.G.-G.); m.patino@oan.es (M.P.-E.); francisco.beltran@oan.es (F.J.B.-M.); m.bautista@oan.es (M.B.-D.); 2 Department of Signal Theory and Communications, Escuela Politecnica Superior, Universidad de Alcala, Campus Universitario, Ctra. de Madrid a Barcelona km 33.600, 28805 Alcala de Henares, Spain; pablo.lopez@uah.es

**Keywords:** VGOS, RAEGE, VLBI, geodesy, radio telescope, cable-cal, receivers, phase, noise

## Abstract

This paper presents the new cable delay measurement system (CDMS) designed at Yebes Observatory (IGN, Spain), which is required for the VLBI Global Observing System (VGOS) stations. This system measures the phase difference between the 5 MHz reference signal from the hydrogen maser and the 5 MHz signal that reaches the broadband receiver through a coaxial cable, for the generation of calibration tones. As a result, the system detects the changes in the length of that coaxial cable due to temperature variations along the cable run and flexures caused by VGOS radio telescope movements. This CDMS outperforms the previous versions: firstly, it does not require a frequency counter for phase/delay measurements; secondly, it largely reduces the use of digital circuits; hence, reducing digital noise; and thirdly, it has a remotely controlled automatic calibration subsystem. The system was tested in the laboratory and in the radio telescope, and the measurements of both set-ups are shown. These measurements include the total noise, accuracy, hysteresis, and stability. The results in the radio telescope can be correlated with the different factors that affect the cable, such as temperature and flexures. The system allows to achieve an RMS noise of less than 0.5 ps, significantly improving the requirements established in VGOS. The system is currently installed in the Red Atlántica de Estaciones Geodinámicas y Espaciales (RAEGE)Yebes VGOS 13.2 m radio telescope, and will be installed in the Norwegian Mapping Authority (NMA) twin VGOS radio telescopes, in the Finnish Geospatial Research Institute (FGI) VGOS station and in the RAEGE Santa María VGOS radio telescope (Açores, Portugal).

## 1. Introduction

Geodesy is the scientific discipline that studies the Earth’s shape, dimensions, orientation, and position in space. This includes the definition of the external gravity field of the Earth, and the surface of the ocean floor.

One of the more accurate techniques to measure such Earth parameters is geodetic very long baseline interferometry (VLBI). This technique computes these parameters from the measurement of radio telescopes base lines, which are derived from coordinated and simultaneous observations of many quasars from different VLBI stations around the world. The signal from a quasar is received in each radio telescope with a different delay, depending of its position on the Earth. The data are recorded with very accurate information of the arrival time using an hydrogen maser clock at each radio telescope. Then, the data are correlated, reduced, and analyzed to determine the desired Earth parameters [1,2].

The Spanish National Geographic Institute (IGN) is involved in the so-called RAEGE [3,4] project together with the Regional Government of Açores (GRA, Portugal). RAEGE stands for Red Atlántica de Estaciones Geodinámicas y Espaciales. The aim of this project is to build, install, and operate four geodetic core sites in different tectonic plates. The locations are Spanish mainland (Yebes, Guadalajara), Santa María and Flores islands (Açores archipelago, Portugal), and Gran Canaria island (Canary Islands, Spain). The radio telescopes of these core sites are compliant with the VGOS project, whose goal is to reach millimeter accuracies in the determination of the baselines between radio telescopes by using fast-moving antennas, sensitive broadband (2–14 GHz) low-noise receivers, and high-throughput backends. These capabilities will allow fast and periodic measurements of the geodetic parameters of our planet with high accuracy [5].

The VGOS project aims to replace the legacy geodetic VLBI system (in S and X bands) where centimeter accuracies in baselines are achieved. To improve this, the VGOS receivers will provide four sub-bands of 1 GHz in width, in two polarizations, across the 2–14 GHz band. The combination of this receiver with fast-moving antennas to observe a large number of sources (quasars), will improve baseline accuracies to the millimeter range. Therefore, the accuracy of VLBI measurements will be improved by a factor of 10 with the advent of VGOS [6].

**Figure 1:**
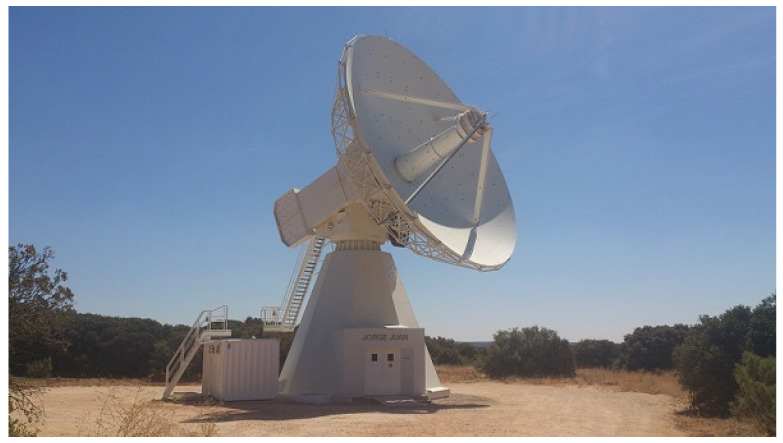
Yebes RAEGE VGOS 13.2 m radio telescope.

Figure 1 shows the Yebes RAEGE VGOS radio telescope equipped with a broadband receiver [7].

**Figure 2:**
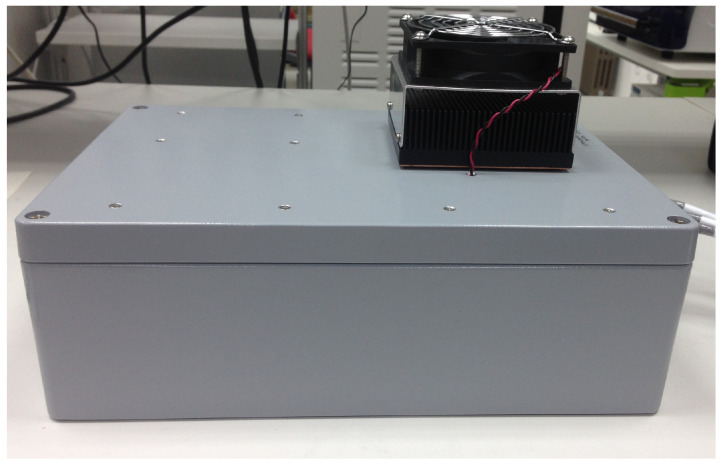
View of a phaseCal antenna unit (AU) enclosure, with a Peltier heat sink and associated fan.

The high accuracy required for VGOS observations make it necessary to use a system that allows the phase calibration of the whole receiver chain. This system is called phaseCal [8,9] and its mission is twofold:

- Inject a phase-stable and low-power reference signal at the receiver input. The signal will be locked to the maser reference. This allows the monitorization of phase changes of the whole receiver system during observations, and the determination of the relative phases between the local oscillators of the baseband converters (BBCs) [10]. For that, the phaseCal system generates, from a 5 MHz signal derived from the hydrogen maser, a series of 5 or 10 MHz equally spaced spectral lines (pulse train). This pulse train is injected in the receiver through a microwave directional coupler placed inside the dewar, just before the cryogenic low-noise amplifier (LNA), and the pulses are detected together with the desired signal from the quasar. This function is performed by the so-called phaseCal antenna unit (AU), located very close to the broadband receiver (Figure 2). This technique allows a correct calibration of the phase delay introduced by the receiver chain, during the data reduction process [11].
- Monitor phase changes produced in the coaxial cable that carries the 5 MHz reference signal from the maser to the phaseCal AU, for the generation of the pulse train. This function is performed by the so-called phaseCal ground unit (GU) or cable delay measurement system (CDMS), located in the backend room, or close to the hydrogen maser. These phase changes are due to temperature gradients along the cable path and cable flexures caused by radio telescope movements. Therefore, the 5 MHz signal delay through this cable varies during the observation time. To monitor this effect, the AU module returns to the GU a sample of the received 5 MHz signal, but modulated by a 5 KHz signal. After demodulation, the 5 KHz signal is compared in the GU, with the 5 KHz from the original reference one, in order to measure the signal delay along the coaxial cable. The modulation is required to distinguish the 5 MHz signal going to the AU from the one returned by the AU, as they travel through the same coaxial cable [12].

This paper will focus on a new phaseCal ground unit design that improves the resolution and accuracy and reduce the required resources to perform the signal processing and delay measurements.

This system is basic in geodesy observations since it allows the removal of instrumental errors caused by phase changes in the reference signal when it is transported from the maser clock to the radiofrequency receiver.

There are different versions of this development. The first and oldest one is the system developed by Alan Rogers [13]. This CDMS was used by many VLBI stations, including the Yebes Observatory, for a long time. However, a new version had to be developed since the system did not meet the requirements imposed by the new VGOS observations.

NASA developed a new version to meet VGOS specifications based on an software-defined radio (SDR) in which the phase of a 5 MHz signal and a 5.001 MHz signal is compared. This new system allows the calibration of the reference signal to be implemented in both coaxial and optical fiber and it is used in other VGOS stations, such as the Onsala one (Sweden) [14,15].

Another version was designed by the Korean VLBI system for Geodesy (KVG) based on the phase difference measurement of two close frequency signals [16].

## 2. Materials and Methods

The new GU is based on the previous version of the Yebes GU, but it solves the issues related with digital noise associated with its digital circuits. It is a version of the design from Alan Rogers [13], but with different modifications that reduce its size, the cost, and simplify its design, keeping the system performance.

It is based on the constant phase comparison of the 5 MHz signal from the maser with the signal that is returned from the AU. In this way, the monitoring of this phase difference provides a measurement of the two-way cable delay. The one-way cable delay of the 5 MHz signal is the half value of the two-way cable delay, as both signals (transmitted and returned) run along the same cable.

In order to distinguish the transmitted and returned 5 MHz signals, the returned one is modulated with 5 KHz. Upon arrival to the GU, the modulation is removed in a phase-locked loop (PLL), which provides a loop voltage proportional to the phase difference between both signals.

The goal is to measure the small phase differences between the two signals with picosecond accuracy. It can be done without frequency conversion, but it would need two high-speed digitizers with the same clock, and a real-time algorithm to compute the delay. Although it is feasible, this approach can be very complex [14].

In the original system, to simplify this measurement, it was decided to convert both signals to a lower intermediate frequency (25 Hz) where their phase differences could be measured with commercial off-the-shelf (COTS) frequency counters. A simple scale factor (5 MHz/25 Hz) is required to translate the measurements at 25 Hz to 5 MHz.

**Figure 3:**
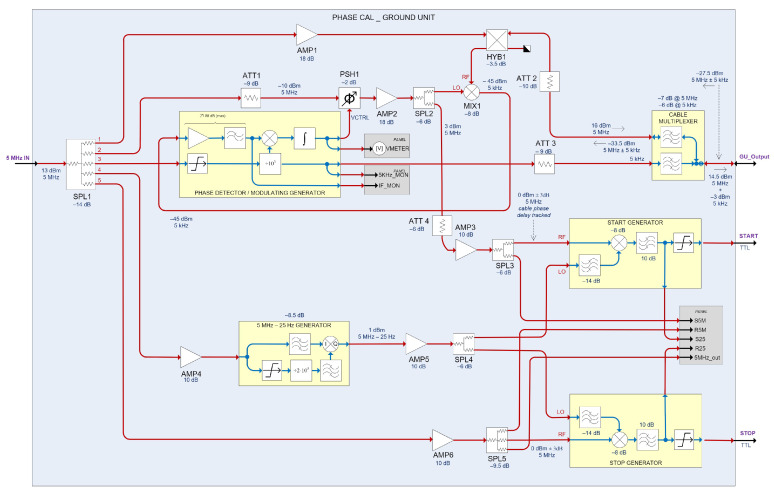
Block diagram of the previous GU designed in Yebes Observatory, and based on the Alan Rogers design.

Figure 3 shows the block diagram of the Yebes GU previous system based on [13].

However, this system has shown some issues during its operation in the 13.2 m radio telescope at Yebes Observatory, which has led to wrong delay measurements. The problem was related to random oscillations of ultra-fast digital comparators, which perform signal conditioning before reaching the frequency counter (START/STOP generators in Figure 3).

Therefore, it was decided to develop a new GU with as few digital circuits as possible.

The main block of the GU is the PLL. This loop generates a phase-shifted replica of the 5 MHz reference signal. The phase-shift applied by the PLL is equal to the phase of the returned 5 MHz signal from the AU, which has performed a two-way travel (round-trip), from the GU to the AU and back.

The proposed new GU determines the phase difference from the measurement of the DC voltage generated by the PLL, which is proportional to the phase difference. This voltage can be measured with high resolution by either a 24-bit ADC’s (18–20 effective bits) with low sampling rates (1 sample/second) or with a high-accuracy multimeter (6.5 effective digits, at least), which provides a very good resolution in the microvolt range.

Therefore, by reading the voltage with high resolution and a subsequent digital signal processing (digital low-pass filter with 10 Hz cut-off frequency), an accuracy in the range of 1 ps is achieved.

The solution adopted for the new system will preserve the phase-locked loop of the original system, and the loop control voltage will be read by a multimeter with 6.5 digits of resolution. Additionally, the phase-locked loop will be magnetically and thermally insulated to prevent possible external effects that may distort the behavior of the ferrites that compose the phase shifter.

**Figure 4:**
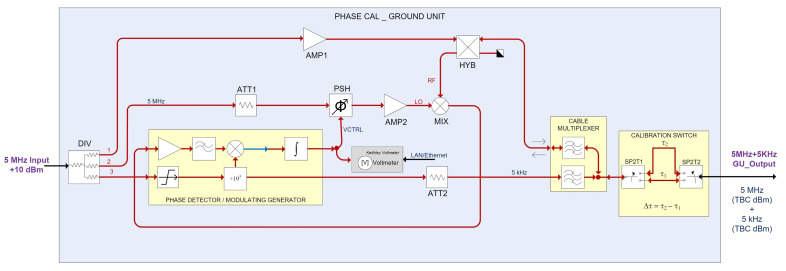
Block diagram of the new GU designed in Yebes Observatory.

The new scheme for the GU is shown in Figure 4, as can be seen, the number of elements has been drastically reduced.

The relationship between phase shift and voltage (volts-to-picoseconds scale factor) will be determined by inserting well-calibrated phase changes in the 5 MHz signal path, when the system operates in its linear range. These changes will result in a certain voltage change and, as a result, the scale factor of the system (mV/ps) will be computed.

For this purpose, two coaxial cables of different lengths switched by two SPDT coaxial switches, as shown in Figure 4. The time delay difference between both paths has been carefully measured with a vector network analyzer (VNA) in the time domain, where femtosecond accuracy is achieved.

Once the system is installed and it is in a thermally stable environment, the calibration will be performed It is also recommended to perform the calibration before each VGOS session. To perform the calibration, the SPDT will be switched introducing the known delay, the voltage-picosecond scale factor is calculated, thus obtaining the delay in picoseconds.

**Figure 5:**
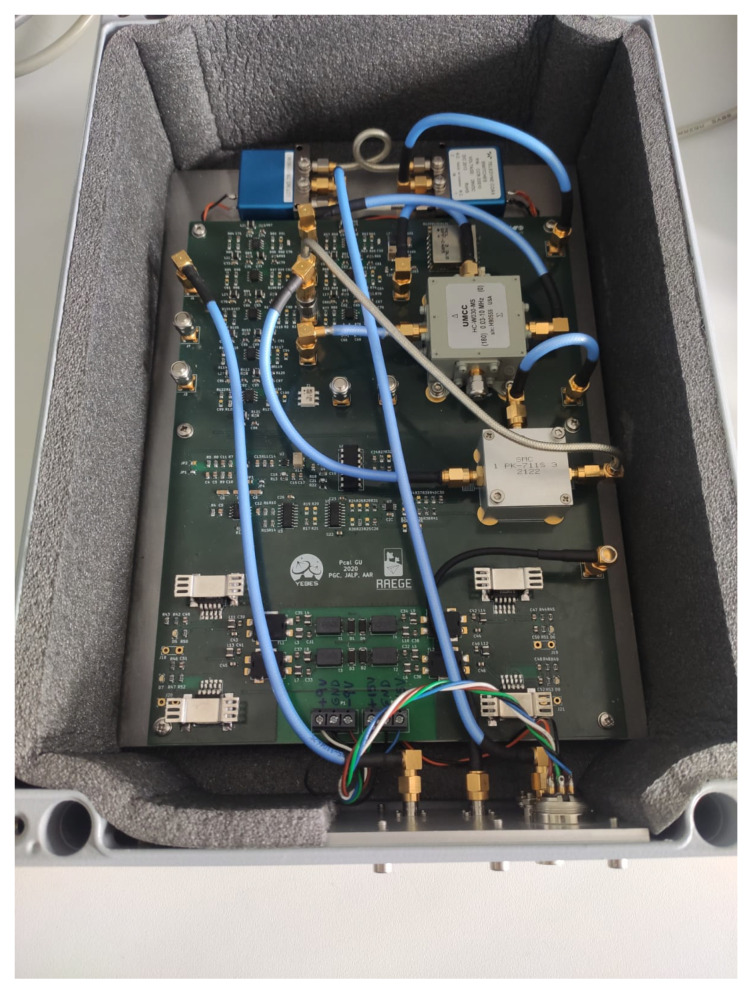
Internal view of phaseCal GU.

**Figure 6:**
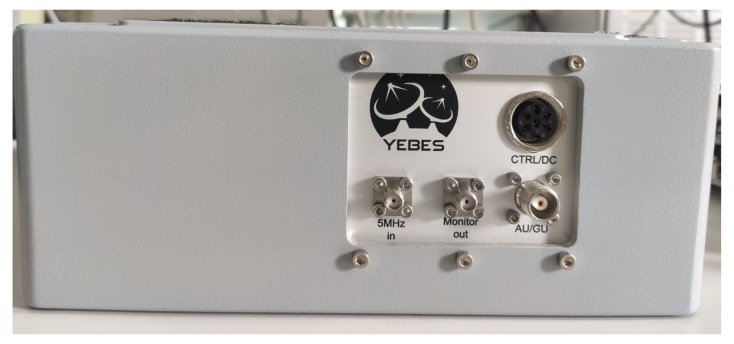
External view of phaseCal GU.

Figure 5 shows the internal view of the new phaseCal GU, with its corresponding printed-circuit board and devices. Figure 6 shows the interface panel of the new GU.

## 3. Results and Discussion

In this section, the results of the measurements on the new phaseCal GU system will be presented. These measurements were carried out in the laboratory and after its installation in the Yebes RAEGE VGOS antenna.

It will also be shown how the good accuracy of the new system reveals the effects on the cable, induced by thermal changes and flexures. This information is critical in order to take actions to reduce or mitigate those effects on the cable.

For the new GU, the following sections will show the accuracy, the linearity, the hysteresis, and the noise of the measurement. The dependence between temperature and cable changes and the effect of flexures in both azimuth and elevation antenna movements will also be shown.

### 3.1. Laboratory Tests

#### 3.1.1. Accuracy

To measure the accuracy of the system, a Narda manual phase shifter was used. It allows the manual insertion of known phase offsets in the 5 MHz signal path, from 0${}^{\circ }$ to 180${}^{\circ }$ at 1 GHz, which corresponds to offsets from 0${}^{\circ }$ to 0.9${}^{\circ }$ at 5 MHz (0.9${}^{\circ }$ at 5 MHz equals 500 ps). Therefore, it is possible to introduce very precise and known delays in the system to verify that the system output reproduces these changes.

**Figure 7:**
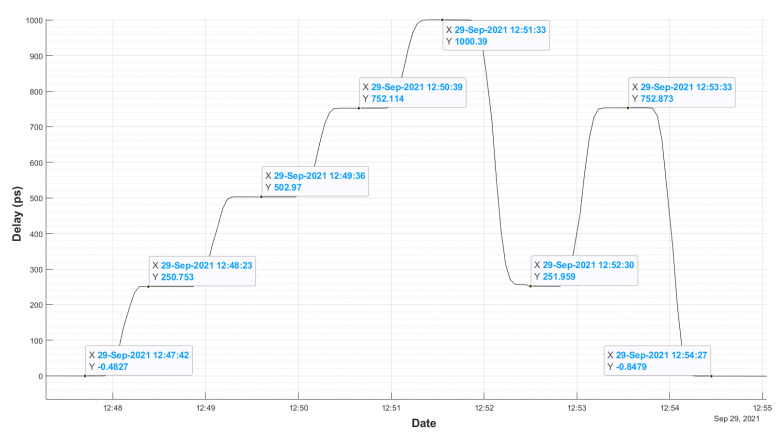
Phase Cal GU response to changes of 250 ps (two-way) controlled by a manual phase shifter.

Figure 7 shows the time evolution of the measured delay when steps of 0, 125, 250, 375, and 500 ps in the phase shifter wheel are applied. As the 5 MHz signal performs a two-way trip from the GU to AU, the total measured delay is twice this value, can be seen in the Figure 7.

**Figure 8:**
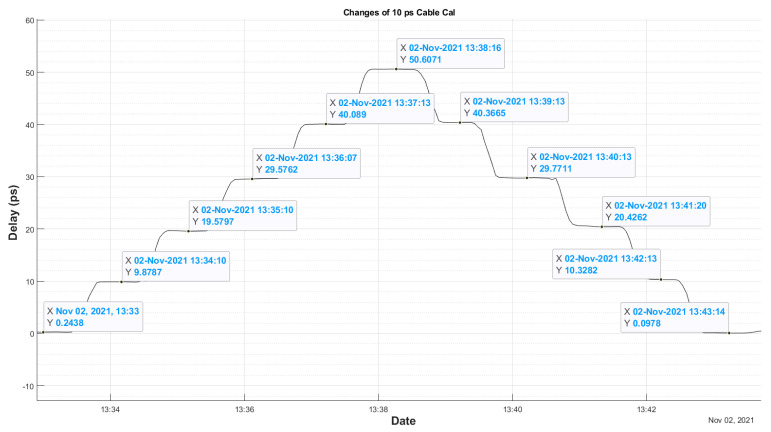
Phase Cal GU response to changes of 10 ps controlled by a phase shifter.

Additionally, Figure 8 shows the time evolution of the measured delay for smaller steps of 10 ps (two-way). It can be seen that these changes are easily detected. However, it must be taken into account that the changes are made using the shifter’s wheel, and the wheel settings can be affected by some slack.

These results show high accuracy, low noise, and good linearity in the range between 0 and 1 ns (the cable is not expected to change more than 300 ps).

#### 3.1.2. Hysteresis

Figure 7 and Figure 8 also show a hysteresis test, because they include cases with phase shifts in increasing and decreasing values. The results are shown in Table 1, and it must be remembered that the phase shift settings can be affected by some slack or play in the shifter wheel.

Table 1 shows the hysteresis of the system. This is less than 1.2 ps. Errors in this measurement may be due to the tolerance in the phase shifter, small drift of the CDMS system, variations in the cable during the time of measurement, in addition to the error in the accuracy of the developed GU.

However, as can be seen, the noise of the system is small enough to reach the specs, the RMS noise of the measured hysteresis is below 0.6 ps.

**Figure 9:**
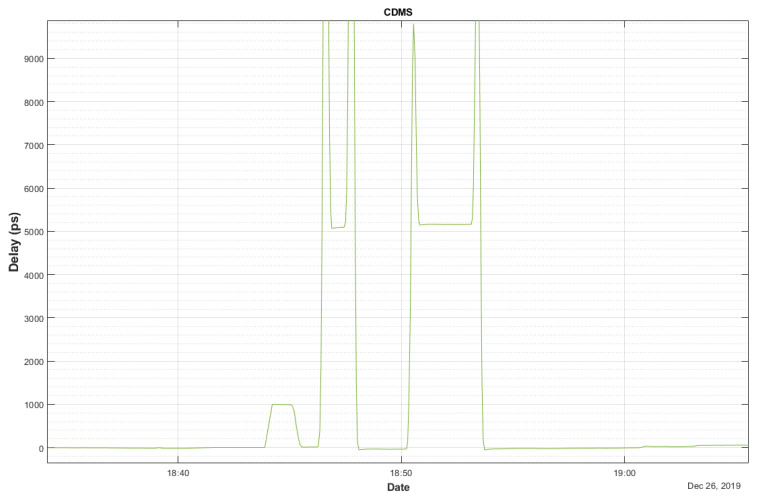
Delay measurements for the phaseCal GU test cable.

In addition to the table, Figure 9 shows the results for a high delay of 5.2 ns, inserted by the phaseCal test cable, which is used at the beginning of each VGOS session to check that the phaseCal GU is working fine.

Some spikes resulting from the connection and disconnection of the test cable can be seen, but when the cable is inserted, it reaches the required value (5200 ns) and when disconnected, it returns to the starting value (0 ns), as expected.

#### 3.1.3. System Noise

The noise of the system provides a value of the measurement error introduced by the system itself. This error is the ultimate accuracy that the system can reach in the absence of the effects on the cable to be measured.

It should be noted that it was not possible to have a perfectly stable thermal environment for this test, so the short cable connection between GU and AU was subject to some temperature drift.

**Figure 10:**
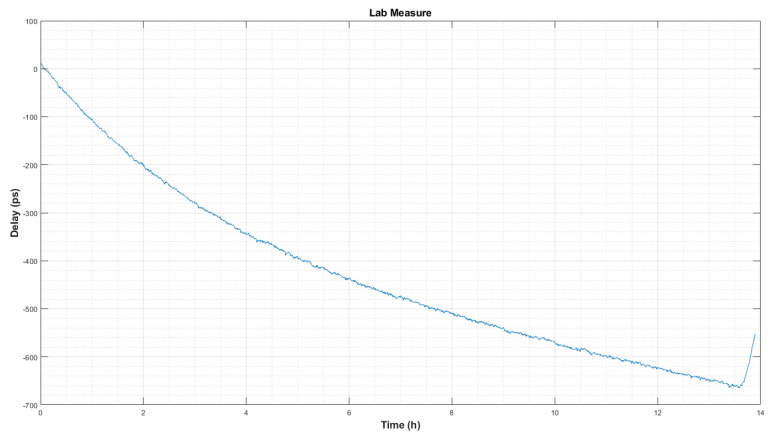
Delay measurement in the laboratory. The slope is due to thermal drift caused by ambient temperature.

Figure 10 shows a measurement of the system in the laboratory with a 3 m cable reel.

It should be taken into account that, in the laboratory, the thermal conditions were changing, as there was other electronic equipment switched on, there was movement of staff, and windows were open due to the COVID-19 restrictions. This is the reason for the 600 ps change along the 13 h long measurement shown in Figure 10.

**Figure 11:**
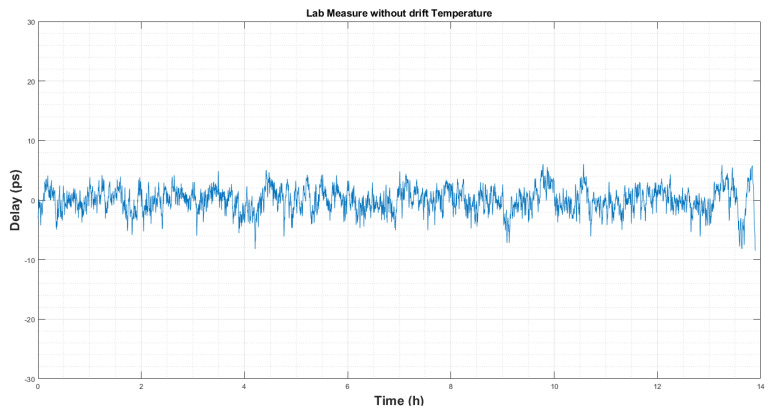
Delay measurement in the laboratory after drift removal by curve fitting.

In order to know the final RMS noise of the delay measurement, a fourth-order polynomial was used to to fit Figure 10 temperature variations. After removing those effects, the remaining noise can be seen in Figure 11.

Therefore, it can be said that, during a 13 h test in the thermal conditions explained above, the final peak-to-peak noise is less than 10 ps, and the computed RMS noise is 2.4 ps, which is a very reasonable value.

In order to improve the thermal environment of the test, the set-up was taken to the Yebes gravimeter room, where the temperature had a gradient of $\pm 1^{\circ }$ only, and a 1 m cable was used instead of the 3 m one.

**Figure 12:**
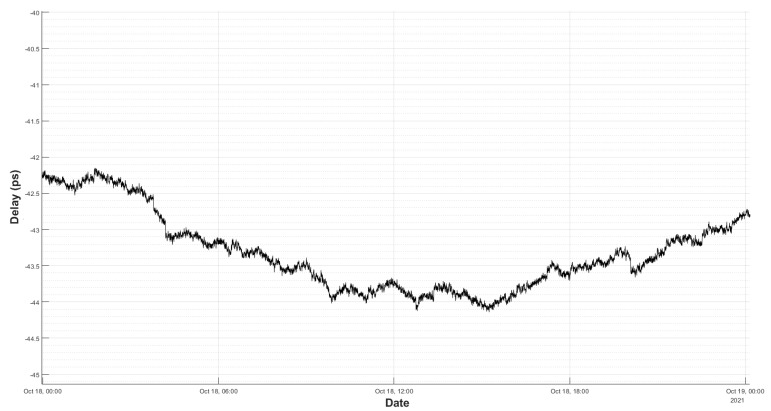
Delay measurement in a thermally stable room for 24 h.

Figure 12 shows the result of this test. It can be seen that, in 24 h, the total drift was around 2 ps and it does not show a dominant slope, as in Figure 10. It can be concluded that the thermal drift in the laboratory impacted the accuracy of the system.

**Figure 13:**
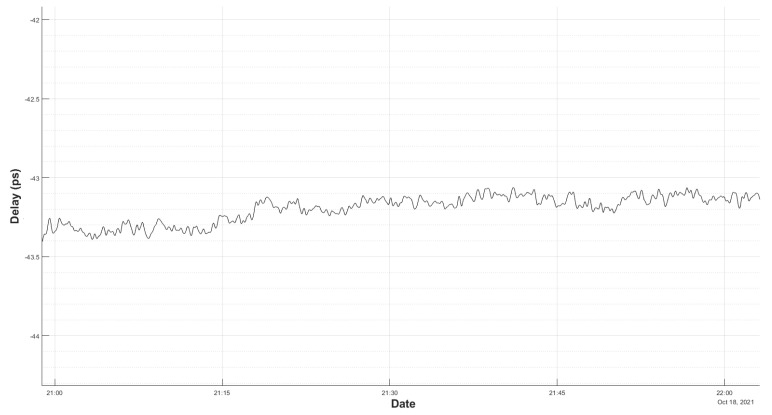
Zoom of 60 min of the delay measurement in a stable room.

**Figure 14:**
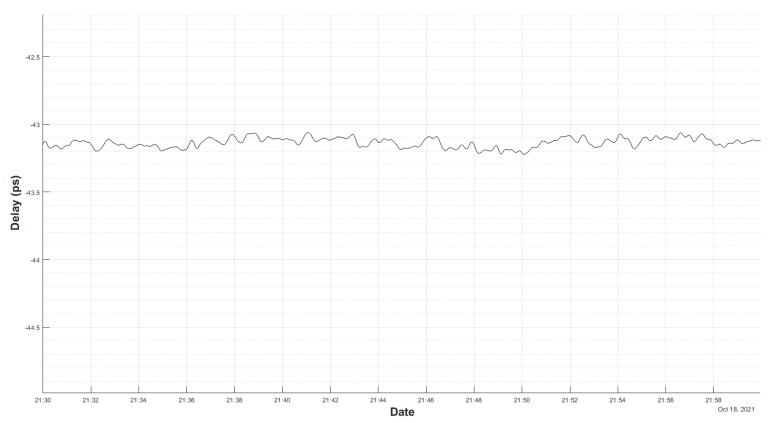
Zoom of 30 min of the delay measurement in a stable room.

Even so, the new environment is not perfect and it will affect the measurements, as the low-frequency ripple in Figure 12 shows. If the RMS noise is computed in different sections (as shown in Figure 13 and Figure 14), the results of Table 2 are obtained.

#### 3.1.4. Allan Variance

The Allan variance (ADEV) [17] was computed from the measurements in order to evaluate the stability of the new GU system. It allows a direct comparison with the VGOS requirement.

**Figure 15:**
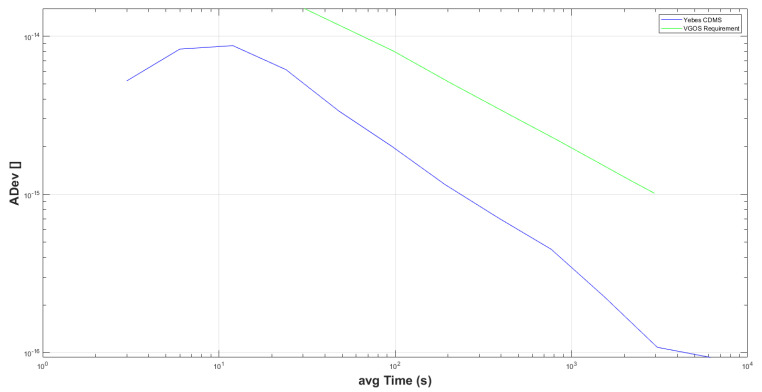
Allan deviation of new GU system in the comparison with VGOS requirements.

Therefore, the ADEV was computed from the data shown in Figure 12, and the results are shown in Figure 15. The blue line shows the ADEV of the new GU system, while the green one shows the VGOS requirement. It can be seen that the new system clearly meets the requirements. For instance, for a typical VGOS 30 s scan time, the requirement is 1.5 $\times \;10^{-14}$ while the system provides $5\times 10^{-15}$.

### 3.2. Tests with the System Installed in the Radio Telescope

Currently, the new phaseCal GU is located in the concrete tower of the RAEGE VGOS radio telescope pedestal.

The 5 MHz reference signal from the maser reaches the radio telescope pedestal via RF-over-fiber links, where a distributor generates four copies. One copy is routed to the phaseCal GU. The GU generates a 5 KHz signal by frequency division of the 5 MHz one, and sends a combination of 5 MHz + 5 KHz through a 50-m coaxial cable towards the phaseCal AU, which is integrated very close to the receiver front-end in the VGOS receiver trolley.

The cable is thermally insulated inside a plastic tube, along most of its route, but it suffers from bending due to the azimuth and elevation movements of the radio telescope. In addition, despite this insulation and the fact that the concrete pedestal has thick walls that keep the temperature stable inside, there are sections of the cable path that run outdoors and, therefore, the thermal gradient in its route can become large.

In the following sections, the results obtained when installing the GU in the radio telescope will be shown, as well as different actions that have been carried out to minimize external effects.

#### 3.2.1. Noise

**Figure 16:**
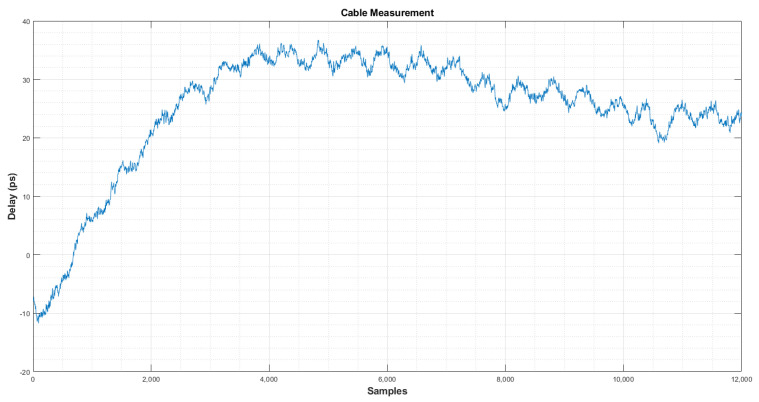
Stability of the system with the radio telescope in stow position.

To measure the noise initially, the system was installed and the corresponding calibration was carried out. After this, a 6 h test during the night (more stable temperature) was measured and the results can be seen in Figure 16. It shows the stability of the system while the radio telescope is in stow position. After an initial slope of 30 ps, the delay oscillates only in a range of 10 ps peak-to-peak.

#### 3.2.2. Flexure and Thermal Effects

**Figure 17:**
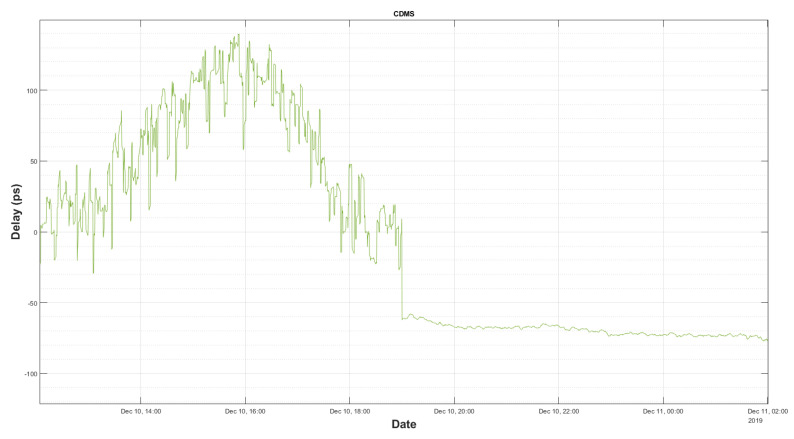
Measured delay during and after radio telescope movement.

Figure 17 shows how the delay in the cable changes along 15 h. During the first half of the time, a VGOS session was underway, and it can be seen that the ripple of the delay increased. This is due to the fact that, although the cable was properly laid-out in its cable trays, it suffered from some flexures in the cable-wraps, as the radio telescope moved in azimuth and elevation. During the second half of the plot, the radio telescope was stopped, as the VGOS session was finished.

**Figure 18:**
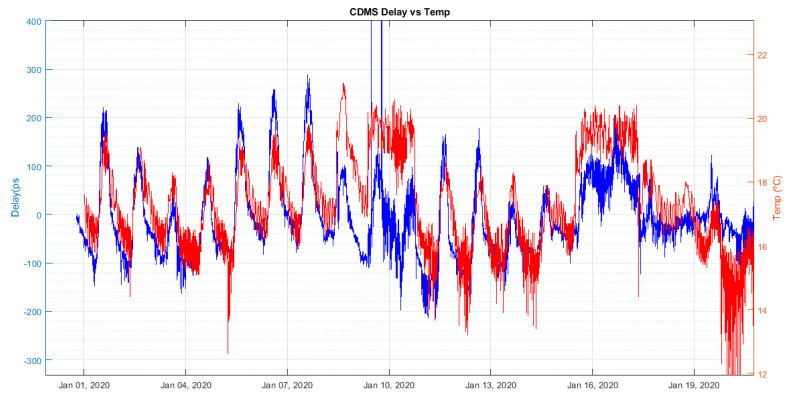
Comparison between receiver room temperature (red) and cable delay (blue).

The daily thermal drift affecting the 5 MHz cable is also observed as a 24-h period ripple. Figure 18, shows how the cable delay (blue trace) and the variations of the temperature in the receiver room (red trace). It was concluded that this ripple in the delay measurements was due to the air conditioning system in the receiver room. This measurement shows that the new GU system was working correctly and detected the changes induced in the cable by temperature fluctuations.

**Figure 19:**
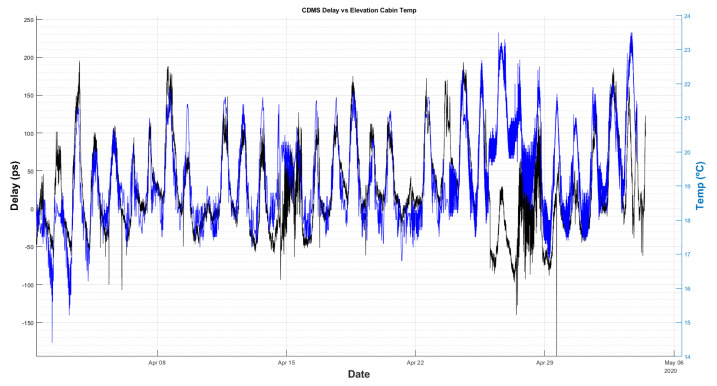
Comparison between delay (black) and receiver room temperature (blue).

**Figure 20:**
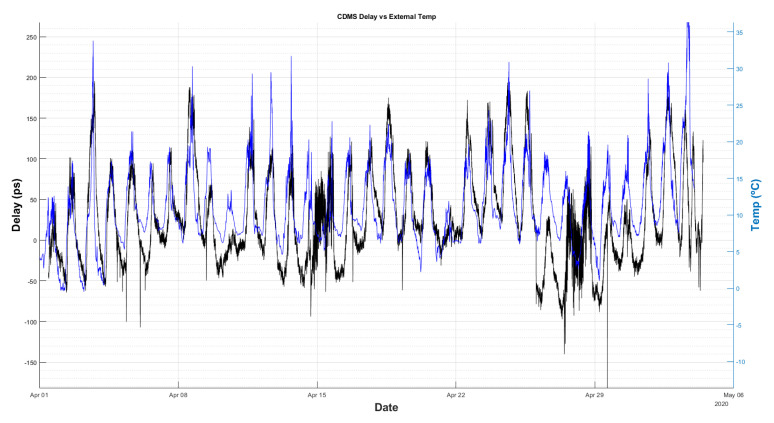
Comparison between delay (black) and ambient temperature (blue).

A longer period of delay measurements was recorded (5 weeks) with the new GU working without any intervention. Figure 19 and Figure 20 compare the cable delay with the temperature of the receiver room and the external ambient temperature, respectively.

From these figures, it cannot be concluded which temperature (receiver room or ambient) is dominant on the delay measurements. Nevertheless, it is important to thermalize the radio telescope receiver room, in order to minimize the impact of its thermal fluctuations on the coaxial cable. This way, it will be revealed which of the two effects is the dominant, so actions can be foreseen to mitigate it.

#### 3.2.3. Azimuth—Elevation Dependence of Delay

This section covers the characterization performed to study the dependence of the delay on azimuth and elevation movements of the radio telescope. The experiment was composed of two parts:

(a) Radio telescope movements from 7${}^{\circ }$ to 87${}^{\circ }$ with 10${}^{\circ }$ steps. After reaching the maximum elevation, it was repeated downwards. It was repeated for each azimuth in the range of 0${}^{\circ }$ to 360${}^{\circ }$ with 20${}^{\circ }$ steps.
(b) Clockwise azimuth sweep from 0${}^{\circ }$ to 360${}^{\circ }$ with 20${}^{\circ }$ steps. It was repeated for elevations from 7${}^{\circ }$ to 87${}^{\circ }$ with 20${}^{\circ }$ steps.

**Figure 21:**
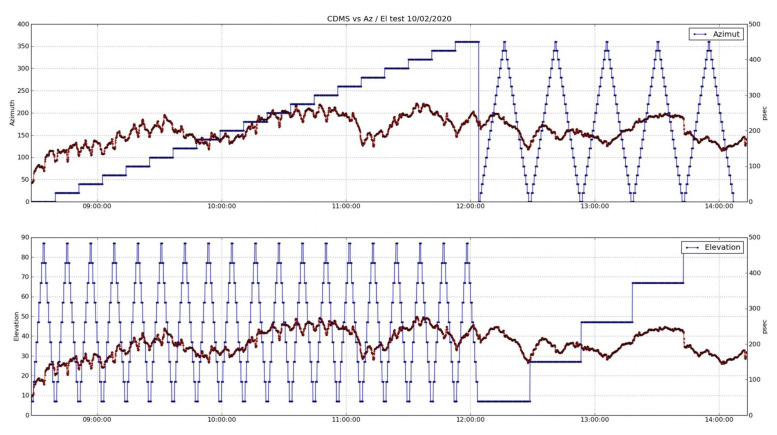
Time evolution of cable delay vs. azimuth and elevation angles.

Figure 21 shows two plots with the delay trace (red) over the azimuth and elevation angles (blue trace in the upper and lower charts, respectively). Although the cable delay measurements show a complex evolution, two different patterns are clearly visible. During the first part of the test, there is a smooth oscillation (probably superimposed over a temperature drift) with an evident ripple. This oscillation vanishes at about 12 h, at the same time than the radio telescopes starts the azimuth drifts.

**Figure 22:**
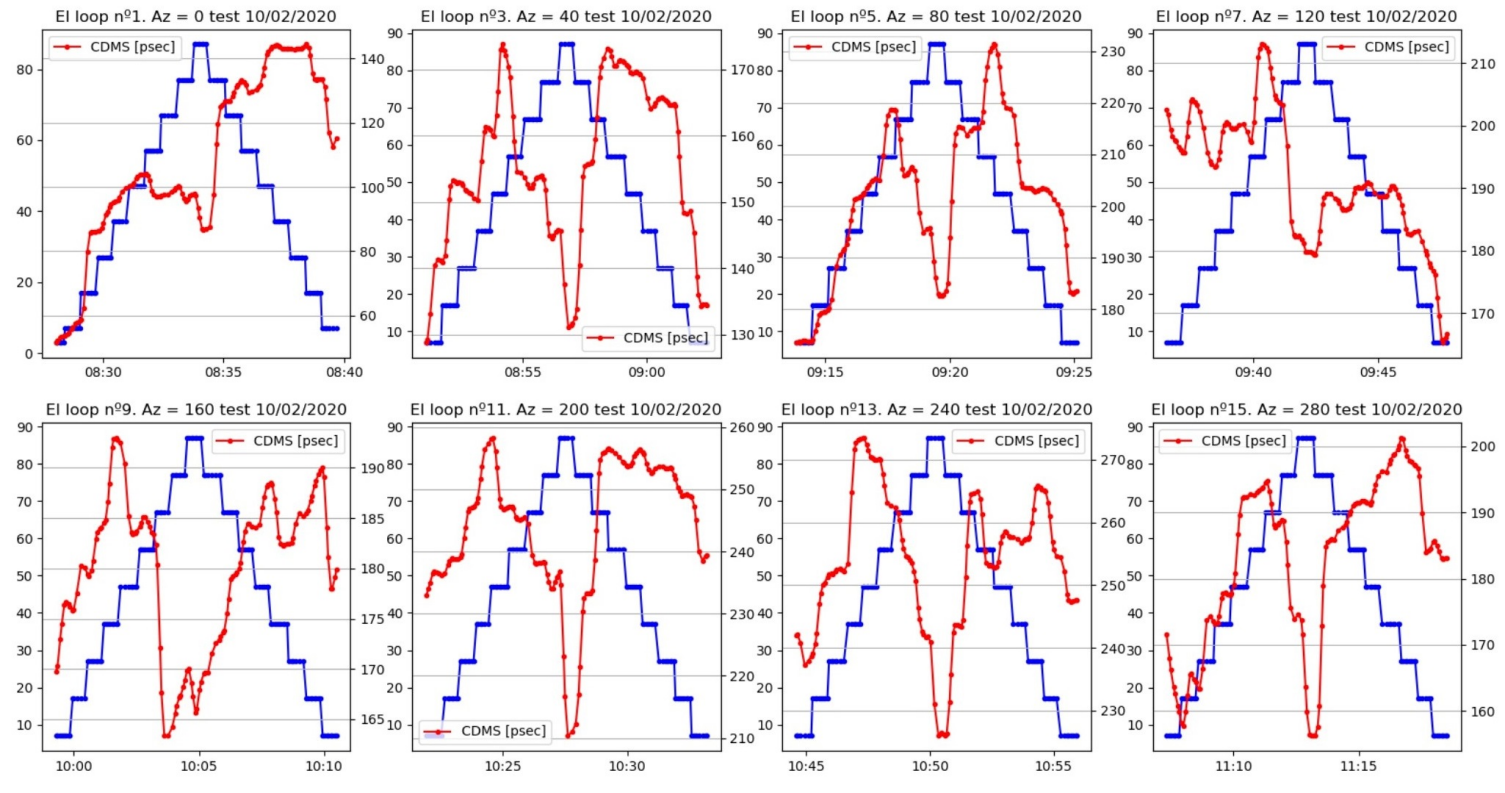
Zoom of the cable delay evolution during elevation loops.

**Figure 23:**
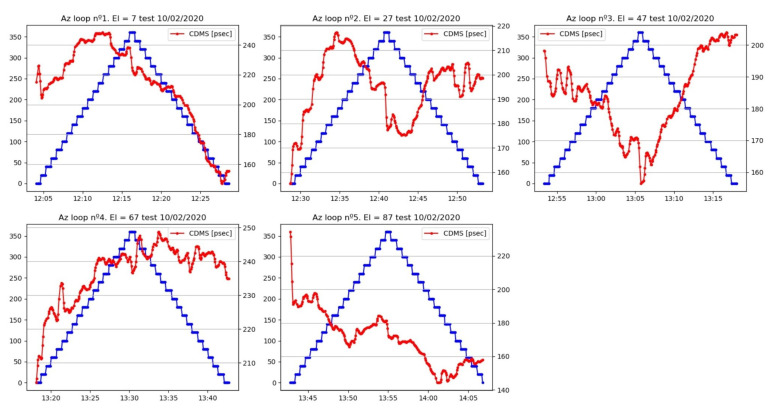
Zoom of the cable delay evolution during azimuth loops.

If a zoom of these plots is selected (Figure 22 and Figure 23), there is a repetitive pattern for the cable delay evolution within the elevation loops. In most cases, when the radio telescope reached angles over 60${}^{\circ }$, approximately, the delay trace dropped, reaching a minimum just before the elevation motion was inverted. After this drop, the cable delay increased until the antenna passed the 60${}^{\circ }$ angle downwards. In this moment, another sudden change of the delay occurred.

Other parameters, such as temperature, which affects the measurement of the cable delay, did not allow us to conclude that there was symmetry in the evolution of the cable during an elevation loop, but we could sense a certain dependence. This dependence seemed to be present in azimuth scans too, although it was not so evident from all of the plots.

**Figure 24:**
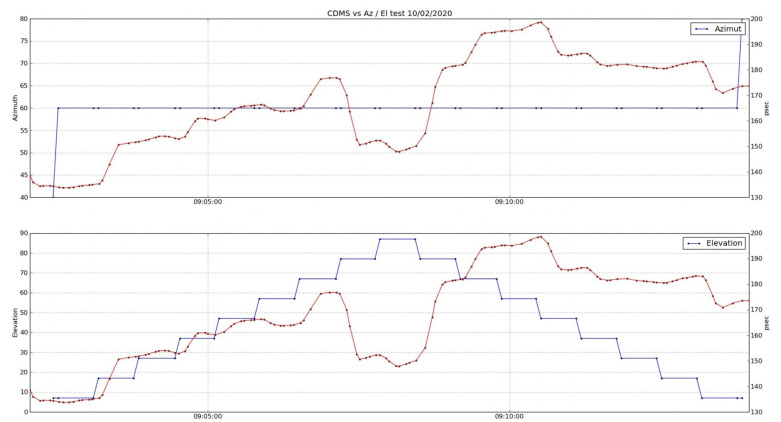
Zoom of cable delay versus azimuth (**top**) and elevation (**bottom**) during the first part of the test.

**Figure 25:**
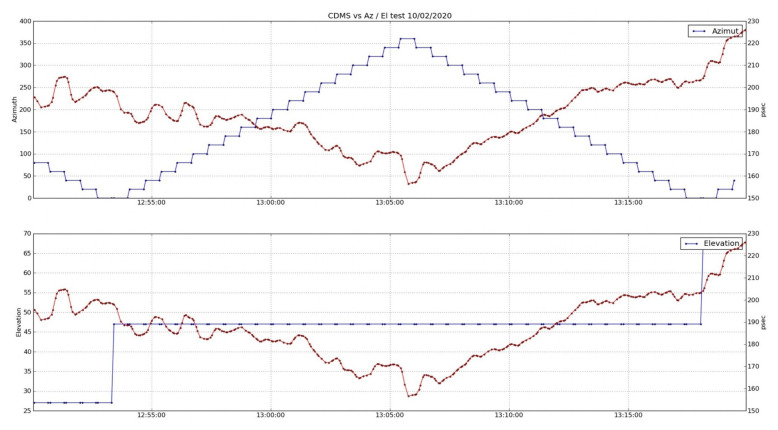
Zoom of cable delay versus azimuth (**top**) and elevation (**bottom**) during the second part of the test.

It is even possible to see a quantization of the changes in the measured cable length for those movement loops with limited noise. This is shown in Figure 24 and Figure 25.

**Figure 26:**
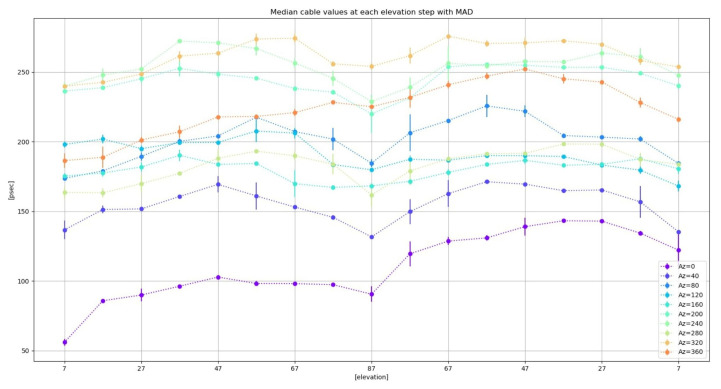
Median values of cable delay measurements at each elevation step for loops at different azimuths. The error bar represents the median absolute deviation (MAD).

**Figure 27:**
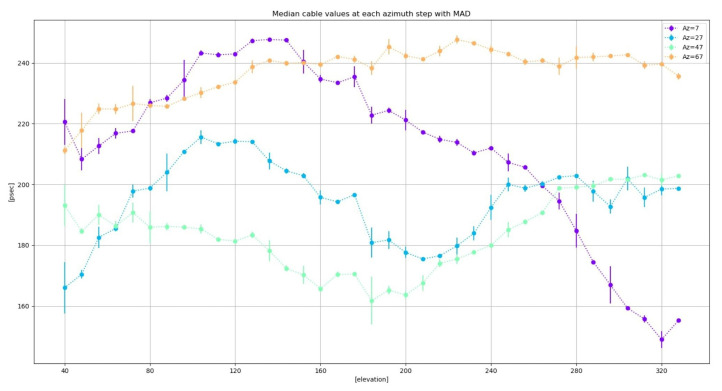
Median values of cable delay measurements at each azimuth step from loops at different elevations. The error bar represents the MAD.

The elevation dependence can be better spotted if the median values of the delay are computed for different elevation steps (see Figure 26). The comparisons between the elevation loops at different azimuths allow the detection of a pattern. In contrast, azimuth sweeps do not show any easily detectable pattern (see Figure 27).

In summary, with the new phaseCal ground unit developed in Yebes Observatory for RAEGE/VGOS radio telescopes, great results have been achieved. The first one is the stability, whose requirement for this system is below 1 ps RMS. This new system shows an improvement by a factor of 2 along 24 h; in shorter times, an improvement of a factor of 10 can be achieved. This is a significant improvement over previous GUs.

Figure 8 shows how the system is capable of reproducing changes of 10 ps quite precisely, taking into account the errors when introducing this delay due to the method used. These results show a behavior comparable to the new system for VGOS developed in Haystack [14].

On the other hand, the new GU meets the ADEV requirements for VGOS, as shown in Figure 15, so it can be implemented in other VGOS stations that require this technology.

However, during tests in the radio telescope, the coaxial cable suffered to some effects induced by both thermal changes and the flexures, during radio telescope movements. These changes were very big, and their impacts on the cable should be mitigated as much as possible.

Regarding flexure changes of the cable, Figure 22 shows a much greater dependence when the antenna moved in elevation than in azimuth. This can be understood by the path of the coaxial cable from the base of the radio telescope to the receiver. However, these phase changes due to the movements of the antenna were too large. As reported by Alan Rogers [18], the changes in this type of coaxial cable (LMR240) should be less than 0.5 ps in bending of a 10 cm radius, a value very far from what was obtained.

Concerning thermal changes, it is necessary to consider whether the dominant effect is inside the receiver cabin or outdoors. To do this, it is required to improve the air-conditioning system of the receiver cabin and to reduce its dependence on the external ambient temperature. Additionally, tests carried out in the laboratory (Figure 10) show that the CDMS system should be placed in a thermally stable location to minimize the possible impact on the PCB board.

As part of future work, we plan to translate this design to a bi-directional fiber optic one, using dense wavelength division multiplexing (DWDM). Optical fiber is more robust than coaxial cable, both to thermal changes and bending, so the delay changes to be measured are supposed to be smaller. In addition, the optical fiber has the benefit of providing an electrical insulation between GU and AU.

## 4. Conclusions

A new phaseCal ground unit system was designed, built, and tested successfully in Yebes Observatory for RAEGE/VGOS radio telescopes.

It is based in the linear relationship between the DC control voltage from a phase-locked loop applied to a 5 MHz analogue phase shifter. This voltage is proportional to the the instantaneous phase changes along the cable path from the GU to the AU.

This system has a resolution of less than 1 ps RMS, and it is based on a simple and cost-effective measurement system (6.5 digit voltmeter), avoiding the use of a frequency counter. It uses a Raspberry Pi single-board computer to implement the digital 10 Hz low-pass filter applied to the voltmeter readings.

It was shown that this new system is much more more stable and accurate than the previous one installed on the RAEGE Yebes 13.2-m radio telescope, and it does not show hysteresis effects.

The system was installed in the RAEGE Yebes 13.2-m radio telescope, and will be installed in the VGOS radio telescopes of the Finnish Geospatial Research Institute (FGI) in Metsähovi, and the Norwegian Mapping Authority (NMA, Norway) in Ny-Alesund (Svalbard).

More stations are expected to have this system in the future, such as the RAEGE Santa Maria station.

## Figures and Tables

**Table 1:** Hysteresis measurements.

Delay	Measured Delay	Measured Delay	Difference
(ps)	(ps in + Direction)	(ps in − Direction)	(ps)
0	−0.48	−0.84	−0.36
250	250.75	251.95	1.2
750	752.11	752.87	0.76
10	9.88	10.33	0.45
20	19.58	20.43	0.85
30	29.57	29.77	0.2
40	40.10	40.37	0.27

**Table 2:** RMS Noise values.

Time Interval	RMS Noise
24 h	0.52 ps
1 h	0.09 ps
30 min	0.04 ps

## Data Availability

Data are available upon request to the correspondence author.

## References

[1] Schuh H., Behrend D. (2012). VLBI: A fascinating technique for geodesy and astrometry. J. Geod..

[2] Nothnagel A., Artz T., Behrend D., Malkin Z. (2017). International VLBI Service for Geodesy and Astrometry—Delivering high-quality products and embarking on observations of the next generation. J. Geod..

[3] Gómez J., Colomer F., López Fernández J.A., Assis M.C.S. RAEGE: An Atlantic Network of Geodynamical Fundamental Stations. Proceedings of the IVS 2010 General Meeting Proceedings.

[4] Vicente P., González J., López-Pérez J.A., Bolano R., Espada S.G., Garcıa P., Beltrán F., Patino M., Garcia O., Serna J.M. The Status of RAEGE. Proceedings of the International VLBI Service for Geodesy and Astrometry 2018 General Meeting.

[5] Petrachenko W.T., Niell A., Corey B.E., Behrend D., Schuh H., Wresnik J. (2011). VLBI2010: Next Generation VLBI System for Geodesy and Astrometry, International Association of Geodesy Symposia. Geod. Planet Earth..

[6] Hase H., Behrend D., Ma C., Petrachenko B., Schuh H., Whitney A. The Emerging VGOS Network of the IVS. Proceedings of the IVS 2012 General Meeting.

[7] García-Carreño P., García-Álvaro S., López-Pérez J.Á., Patino-Esteban M., Serna J.M., Vaquero-Jiménez B., López-Fernández J.Á., López-Espí P., Sánchez-Montero R. Geodetic VLBI ultra low noise broad-band receiver for 13 m VGOS radiotelescopes. Proceedings of the 2016 11th European Microwave Integrated Circuits Conference (EuMIC).

[8] Vicente P., Gallego J.D. Phase Cal: Control Unit. OAN Technical Report 1995-10. https://icts-yebes.oan.es/reports/reports.php?year=1995.

[9] Niell A., Barrett J., Burns A., Cappallo R., Corey B., Derome M., Eckert C., Elosegui P., McWhirter R., Poirier M. (2018). Demonstration of a broadband very long baseline interferometer system: A new instrument for high-precision space geodesy. Radio Sci..

[10] Tuccari G., Alef W., Bertarini A., Buttaccio S., Comoretto G., Graham D., Neidhardt A., Platania P.R., Russo A., Roy A. DBBC VLBI2010. Proceedings of the IVS 2010 General Meeting.

[11] Petrov L. Instrumental errors of geodetic VLBI. Proceedings of the International VLBI Service for Geodesy and Astrometry 2000 General Meeting.

[12] Nothnagel A., Nilsson T., Schuh H. (2018). Very Long Baseline Interferometry: Dependencies on Frequency Stability. Space Sci. Rev..

[13] Rogers A.E.E. (1980). Phase and Group Delay Calibration of a Very Long Baseline Interferometer by East Coast VLBI Group. NASA. Goddard Space Flight Center Radio Interferometry. https://ntrs.nasa.gov/citations/19800020318.

[14] Rajagopalan G. Phase Cal Basics. Proceedings of the 11th IVS Technical Operations Workshop.

[15] Haas R., Casey S., Elgered G., Hammargren R., Helldner L., Hobiger T., Wennerbäck L. Status of the Onsala Twin Telescopes—One Year After the Inauguration. Proceedings of the IVS 2018 General Meeting.

[16] Oh H., Kondo T., Lee J., Kim T., Kim M., Kim S., Ju H. Round-trip System Available to Measure Path Length Variation in Korea VLBI System for Geodesy. Proceedings of the IVS 2010 General Meeting.

[17] Allan D.W. (1966). Statistics of Atomic Frequency Standards.

[18] Rogers A.E.E. (2008). Measurements of Cable Delay with Temperature and Flexure. MARK 5 MEMO #066. https://www.haystack.mit.edu/wp-content/uploads/2020/07/memo_mark-5_066.pdf.

